# Evidence of Woodland Management at the Eneolithic Pile Dwellings (3700–2400 BCE) in the Ljubljansko Barje, Slovenia?

**DOI:** 10.3390/plants12020291

**Published:** 2023-01-07

**Authors:** Welmoed A. Out, Kirsti Hänninen, Maks Merela, Anton Velušček, Caroline Vermeeren, Katarina Čufar

**Affiliations:** 1 Department of Archaeological Science and Conservation, Moesgaard Museum, Moesgaard Allé 20, 8270 Højbjerg, Denmark; 2 BIAX *Consult*, Symon Spiersweg 7d2, 1506 RZ Zaandam, The Netherlands; 3 Department of Wood Science and Technology, Biotechnical Faculty, University of Ljubljana, Jamnikarjeva 101, 1000 Ljubljana, Slovenia; 4 Institute of Archaeology, Research Centre of the Slovenian Academy of Sciences and Art, ZRC SAZU, Novi trg 2, 1000 Ljubljana, Slovenia

**Keywords:** Ljubljansko barje, Slovenia, pile dwellings, Eneolithic, dendroarchaeology, woodland management, coppicing and pollarding, age/diameter analysis

## Abstract

It is assumed that people practiced woodland management, i.e., coppicing and pollarding, in prehistory, but details are poorly known. This study aims for a better understanding of woodland exploitation through time in the wetland basin of the Ljubljansko barje, Slovenia, from 3700–2400 BCE (Before Common Era). To do so, uncarbonized, waterlogged wood from 16 Eneolithic pile dwellings situated in two geographical clusters that cover a time span of c. 1300 years were subjected to age/diameter analysis. It is the first time that age/diameter analysis has been applied to multiple sites from the same region. The investigated posts represent a wide range of taxa, but oak (*Quercus* sp.) and ash (*Fraxinus* sp.) represent 75% of the total, indicating selective use of wood for this purpose. Diameter selection of ash may have taken place as well. At both site clusters, the age/diameter data do not reveal any unequivocal evidence for woodland management. Only at the youngest sites do the data possibly show some gradually changing practices. The outcomes are discussed within the framework of recent discussions about woodland management in Europe.

## 1. Introduction

### 1.1. Woodland Management in Slovenia in the 4th and 3rd Millennium BCE?

It is often suggested that people in Europe practiced woodland management, i.e., pollarding and coppicing, at least from the Neolithic onwards [1], [2] (p. 106). This is also the case for the Circum-Alpine region. For various sites north of the Alps, dendrotypological analyses have indicated that woodland management was practiced in the 4th millennium BCE [3,4], [5] (p. 143), [6]. However, recent studies that investigated wood from seven Neolithic sites dating to the late 5th and 4th millennium BCE in Spain, Denmark (DK), the Netherlands (NL), Slovenia and Sweden using the analysis of branch/trunk age and diameter did not reveal evidence of woodland management at any of the sites [7,8], thus questioning the assumption that woodland management was commonly practiced at that time. Yet these seven case studies all concerned single sites per region and mostly, but not exclusively, single occupation periods, which raises questions about long-term practices. When defining woodland management as the long-term influence of people on trees and shrubs to improve and optimize the quantity and quality of the wood [9], it can be argued that conclusions about woodland management will be stronger when supported by data from multiple sites in a region and/or from longer periods. This paper offers the first possibility of studying woodland management diachronically by age/diameter analysis at multiple sites in a limited region. It concerns 16 Eneolithic pile dwellings dating to 3700–2400 BCE in the southern part of the wetland basin of the Ljubljansko barje (Ger. Laibacher Moor), Slovenia, with all but one located in two geographical clusters (presented in further detail below).

The large number of piles at the 16 pile dwellings suggests that people had access to plenty of wood during the Eneolithic, but it is less clear whether people used particular selection strategies when collecting the wood. Therefore, the aim of this paper is to obtain a better understanding of woodland exploitation at the various Eneolithic sites in the Ljubljansko barje. Questions include which woody plants were used, whether the wood was obtained from managed woodland, whether woodland exploitation strategies were the same for all sites, whether there are any differences between the two occurring site clusters (subregions) within the region and whether there are any changes over time. It has previously been suggested that ash (mainly *Fraxinus excelsior*) may have been obtained by means of coppicing [10] (p. 2037). There are also occasional observations of suppressed growth that may result from shredding. However, an initial age/diameter analysis of ash and oak (*Quercus* sp.) from one of the sites (Stare gmajne) has not provided evidence of management [7]. To investigate this further, all sites in the region for which sufficient relevant data are available were subjected to age/diameter analysis to investigate woodland management. Stare gmajne is again included, this time with more data.

### 1.2. Eneolithic Occupation in Ljubljansko Barje

**Figure 1:**
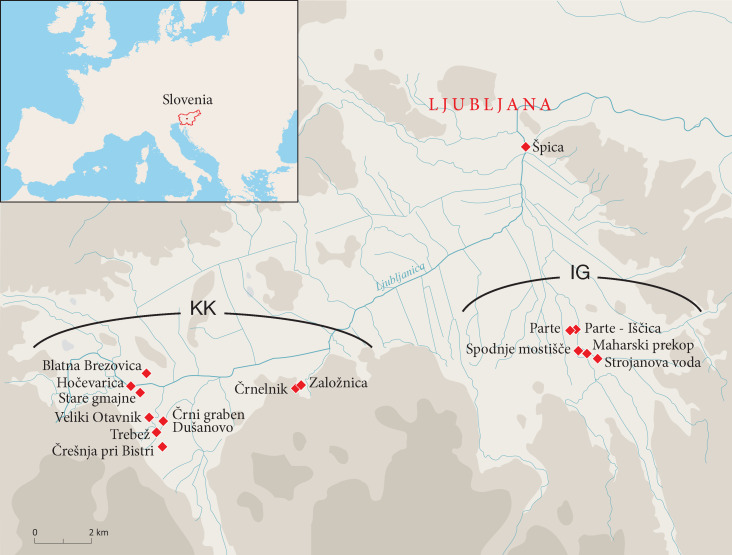
Ljubljansko barje, Slovenia, located southeast of the Alps and directly south of the city of Ljubljana, with the locations of the 16 investigated Eneolithic pile dwellings. Geographical clusters: KK = Kamnik pod Krimom; IG = Ig. The sites Črni graben and Dušanovo represent two excavations of what later has been interpreted as a single site, Dušanovo. After a figure by T. Korošec.

The Ljubljansko barje is a shallow floodplain of 163 km^2^ south and southwest of Ljubljana in Slovenia, southeast of the Alps. From the Late Pleistocene to c. 1000 BCE, the area was covered by a shallow lake that presumably gradually shrank during the Holocene. Currently, the basin is progressively drying out and only occasionally flooded. The vegetation in the middle Holocene presumably consisted of silver fir-beech (*Abies*-*Fagus*) woodlands in the nearby mountains and mixed deciduous woodlands including oak on dry terrain closer to the settlements [11,12]. In this basin, c. 30 pile dwelling sites have been recorded [13]; see also [14] for the research history of the region. The first excavations in the area took place in 1875–1877 [15]. Excavations that included sampling for dendrochronology, palynology, archaeobotany and archaeozoology, among others, were carried out by the Institute of Archaeology, Scientific Research Centre of the Slovenian Academy of Science and Arts in cooperation with the Department of Wood Science and Technology, Biotechnical Faculty, at the University of Ljubljana. These studies concerned small-scale rescue excavations and test trenches carried out on previously excavated areas, riverbeds and in drainage ditches that usually concerned a transect through the sites. Within this framework, samples of uncarbonized, waterlogged wood, mainly from the posts, were systematically collected from 16 Eneolithic pile dwellings (Figure 1, Table 1).

The Eneolithic pile dwellings from the Ljubljansko barje included in this study cover the time period between c. 3700 and 2400 BCE [16,17]. Several cultural horizons are distinguished for this period, including the Furchenstich pottery culture (3700–3550 BCE), the Stare gmajne cultural group (3490–3070 BCE) and the Vučedol culture complex (2490–2420 BCE) (14). Intermittent occupation dates to three main periods: between 3700 and 3330 BCE, 3160 and 3070 BCE, and 2800 and 2400 BCE [18]. Occupation usually took place contemporaneously at different sites. Sites were in use for from 20 to 80 years. Since dating is based primarily on dendrochronology which defines the years trees were felled, construction phases rather than precise occupation phases have been dated (Table 1). At some sites, most of the wood was felled at the same time, while at Strojanova voda, Hočevarica, Stare gmajne, Parte-Iščica and Založnica, multiple building phases have been recognized. Apart from the Špica site, the investigated settlements are located in the southern part of the Ljubljansko barje and form two clusters at a distance of about 10 km from each other—one at Kamnik pod Krimom (KK) with ten sites and one at Ig (IG) with five sites (Figure 1). Concerning vegetation, it is possible that people in each cluster had their own exploitation area that covered both the floodplain and the hills.

Previous studies have placed the onset of local metallurgy in the Ljubljansko barje in the first half of the 4th millennium BCE [18,19,20]. Subsistence at the sites was based on both arable farming and hunting, gathering, fishing and fowling. The main crop plants in the area are emmer wheat (*Triticum turgidum* ssp. *dicoccon*), einkorn (*Triticum monococcum*), six-rowed naked barley (*Hordeum vulgare* var. *nudum*), opium poppy (*Papaver somniferum*), flax (*Linum usitatissimum*) and pea (*Pisum sativum*). Gathered plants include field mustard (*Brassica rapa*), white goosefoot (*Chenopodium album*), acorn (*Quercus* sp.), hazelnut (*Corylus avellana*), water chestnut (*Trapa natans*), apple/pear (*Malus sylvestris/Pyrus pyraster*), dogwood (*Cornus sanguinea*), cornelian cherry (*Cornus mas*), wild grape (*Vitis vinifera* ssp. *sylvestris*), wild strawberry (*Fragaria vesca*) and blackberry (*Rubus fruticosus*), e.g., [21,22,23]. Domesticated and hunted animals include cattle (*Bos taurus*), pig (*Sus scrofa*), sheep (*Ovis aries*), goat (*Capra hircus*), red deer (*Cervus elaphus*), roe deer (*Capreolus capreolus*), chamois (*Rupricapra rupricapra*), various fur animals and birds and fishes [18,23,24,25,26]. One of the sites, Stare gmajne, revealed the oldest wheel in the world, made of ash and oak wood [27]. Some of the sites have yielded evidence of dairying [28,29]. At certain sites, differences between the houses and related find assemblages point to specialization within the prehistoric communities [25]. Occupation at all sites is interpreted as permanent based on indications of cereal cultivation during spring, collection of fruits during late summer/autumn and animal exploitation during winter (e.g., Črnelnik, Hočevarica, Stare gmajne, Blatna Brezovica [22,24,30,31]). In addition, at multiple sites, the trees used for the houses were mainly cut after the growing season, e.g., in autumn, winter or early spring (Čufar and colleagues, unpublished data).

## 2. Materials and Methods

### 2.1. The Ljubljansko Barje Data

As part of the dendrochronological analyses at the 16 Eneolithic sites included in this study, wood slices were cut from uncarbonized, waterlogged wood finds. Due to the sometimes small-scale character of the excavations, the sites were only partially excavated. Almost all wood from the trenches was collected. Apart from Špica, Črnelnik and Veliki Otavnik, all sites were excavated by the same core team. Apart from c. ten artefacts, the wood finds concern either vertical posts that functioned as construction material or redeposited posts for which the original position is unclear.

For the analysis of the taxa, data of 8809 posts that were investigated between 1995 and 2017 were included. Diachronic patterns in the taxon composition per site were not considered because of the different sample sizes. When comparing the clusters, it should be realized that the studied period includes many centuries of intermittent occupation that did not always necessarily take place in both clusters contemporaneously.

For the diameter and age/diameter analysis, the focus was on taxa for which there were reference data available and, to assure sufficiently robust data, for which there were at least 50 measurements of age and diameter available per site. One data set of 48 posts was also included. As a result, the compilation includes data of ash and/or oak from 14 sites. Per site with ≥50 measurements of these taxa, 75–100% of the posts yielded usable age/diameter data (Table 1), representing 4307 ash posts and 1876 oak posts. In addition to ash and oak, data sets of willow from Špica and Založnica were analyzed (*n* = 55 and *n* = 134 respectively). Willow was selected because of the availability of sufficient archaeological data as well as reference data of modern trees.

The archaeological context of the wood, i.e., whether posts were part of the same structure, was not taken into consideration during the age/diameter analysis since this is mostly unknown due to the character and scale of the excavations (see above). Instead, the data of sites that underwent multiple excavation campaigns were initially analyzed per excavation year. When there was overlap between the data of the various campaigns at individual sites, these data were combined (all sites excavated during multiple years, see Table 1). The sites of Črni graben and Dušanovo, resulting from two excavation campaigns, are treated as two sites in this paper but in fact form part of the same complex, Dušanovo. Similar to context, the individual occupation phases to which some posts from Hočevarica and Parte-Iščica have been assigned were also not taken into consideration, since this would result in working with too-small and fragmented datasets (<50 posts).

As for the age/diameter data, four categories of posts were applied:

- Complete round wood with both the pith and the waney edge (latest annual ring) present;
- Incomplete roundwood with the pith and/or the waney edge missing (or not recorded, see assumptions below);
- Complete radially split wood with both the pith and the waney edge present;
- Incomplete radially split wood with the pith and/or waney edge missing (or not recorded, see assumptions below).

Due to the long period of data collection, the information upon which the classification above is based was not always documented explicitly for each individual post. Two assumptions were made:

- Posts for which information about the pith and/or waney edge was missing were classified as incomplete. These assumptions are conservative, and the assemblage presumably contained more complete posts than is documented;
- Posts for which there was no information about whether the wood was split were interpreted as roundwood.

In the results, the few data of complete split wood are grouped with incomplete split wood, since preliminary analysis showed that these data sets overlap each other. For incomplete wood, it is not clear if any annual rings are missing and how many. The age and diameter may thus be larger than measured (in the graphs: potentially moving away from management, in an upwards direction and to the right).

### 2.2. Age/Diameter Analysis

**Figure 2:**
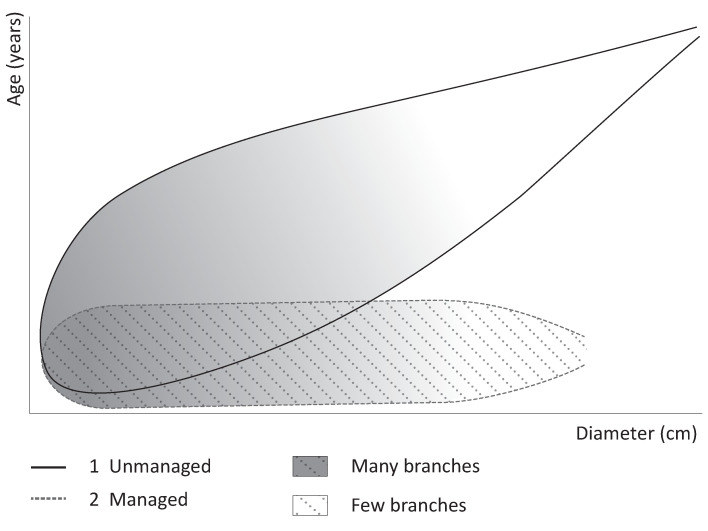
Model for a selection of roundwood and wood derived from roundwood from (1) unmanaged trees, (2) managed trees. This figure is a slightly adapted version of a figure that was published in [9], copyright Elsevier.

The age/diameter analysis to investigate woodland management is based on the use of a model that predicts the relationship between the age and diameter evident in roundwood of unmanaged, natural trees, as well as in coppiced and pollarded trees, i.e., managed trees (Figure 2). The model assumes that, due to management practices, the wood of managed trees grows relatively fast and displays an abrupt end to the age distribution, resulting in a horizontally orientated data cloud in a scatterplot. The model has been tested with roundwood age/diameter analysis of modern unmanaged, pollarded and coppiced trees of several taxa in NL and DK, including ash (78 trees, 2227 measurements) [9,32]. Testing the model for oak with the analysis of modern oak (*Q. robur*) from NL, DK and Germany is ongoing. The age/diameter data from the modern trees of the investigated taxa, based on measurements collected every 1 m of all branches > 1 m, support the validity of the model, showing that roundwood from unmanaged trees with a diameter from 4 to at least 23 cm can be distinguished from that from both coppiced and pollarded trees, particularly in the case of willow and ash. For wood with a diameter < 4 cm, there is much overlap between managed and unmanaged trees, and only very slow growth of unmanaged trees can be detected. There are, in addition, several conditions that should be taken into account:

1. Roundwood age/diameter data of (mostly) free-standing unmanaged trees, benefitting from good access to light and nutrients, show fast growth. They plot in a middle group between the data for unmanaged and managed trees and can, in certain cases, show similarity to the data of managed trees;
2. Natural spurts of unmanaged trees of particularly hazel and willow show fast growth and, as a result, their age/diameter data also plot in a middle group and may overlap with age/diameter data for managed trees. This has also been observed for one modern ash tree but is highly exceptional for this species;
3. The annual ring width of managed trees with a long management cycle declines with time since access to light diminishes. For example, ash trees with a very long management cycle (>50 years) plot as unmanaged trees.

To get a reliable sample, data sets of at least 50 measurements are required in the case of archaeological wood. This is because wood growth may vary depending on environmental conditions such as light, nutrients and herbivory [9]. This also means that instead of single data points, data clouds should be considered for interpretation. It is not essential which part of a tree is sampled.

The model and its limitations are described in detail in Out et al. [9,32]. Until now, the method has only been applied to roundwood, but in this study it was also applied to construction wood derived from roundwood, i.e., radially split wood and posts of which some annual rings may be missing.

To interpret the archaeological data by means of the model presented above, the data of all archaeological case studies have been plotted in scatter plots against a representative selection of modern reference data of the same taxon. The complete reference ash data set consists of 2227 measurements from ≥78 trees from 19 different locations in NL and DK. The complete oak reference data set consists of 1478 measurements from 65 trees in 15 locations in NL, DK and Germany. The complete willow reference data set consists of 2056 measurements from ≥36 trees from 15 locations in NL and DK. In this paper, the following selection is used:

- Average-aged unmanaged trees growing under normal conditions (ash: 7–9 trees, *n* = 159 measurements; oak: three trees, *n* = 204; willow: 10 trees, *n* = 679);
- Old unmanaged trees growing under normal conditions (ash: one tree, *n* = 82; oak: two trees, *n* = 147);
- Average-aged unmanaged trees growing under good conditions because they were (almost) free-standing (ash: nine trees, *n* = 254; oak: four trees, *n* = 193; willow: five trees, *n* = 183).
- Managed trees (ash: 19 trees, *n* = 294; oak: c. 41 trees, *n* = 137; willow: 18 trees, *n* = 714).

Concerning average-aged unmanaged ash growing under good conditions, data from six trees (another *n* = 170 measurements) from branches with a diameter <three cm were not used to increase the readability of the graphs in the overlap area. As for managed ash, data from four trees (*n* = 413) with a diameter <three cm were not used.

An assumption underlying the comparison of reference trees in NL and DK and archaeological data from Slovenia is that the growing conditions in both regions affect tree growth sufficiently similarly to permit comparison. While subtle differences in the growth speed of trees in Slovenia cannot be excluded due to the different climatic conditions, it is expected that the similarities are sufficiently large to allow for the application of the general method and model.

## 3. Results

### 3.1. Taxon Diversity and Woodland Composition

The wood identifications of 8809 posts have shown that the regional woodland vegetation comprised a wide range of taxa (Table 2 and Table S1, based on e.g., [10,16,17,33]). The most common species in the pile assemblages were ash (*Fraxinus excelsior)* and pedunculate/sessile oak (*Quercus robur*/*petraea*), followed by alder (*Alnus glutinosa*), maple (*Acer campestre/pseudoplatanus*), beech (*Fagus sylvatica*), willow (*Salix* sp.), poplar (*Populus* sp.), hornbeam (*Carpinus betulus*), silver fir (*Abies alba*), hazel (*Corylus avellana*) and elm (*Ulmus* sp.). Other taxa were retrieved in small quantities (each <10 in total) and/or were not always optimally preserved and were therefore left out of consideration.

Potential wetland taxa, such as alder, willow and poplar, presumably grew along the lake shore, while ash, elm, maple and pedunculate oak may have grown in or along occasionally flooded zones. Taxa such as beech, maple, hornbeam, sessile oak, elm and silver fir presumably formed the main vegetation elements in the hills that surrounded the lake [12,22].

**Figure 3:**
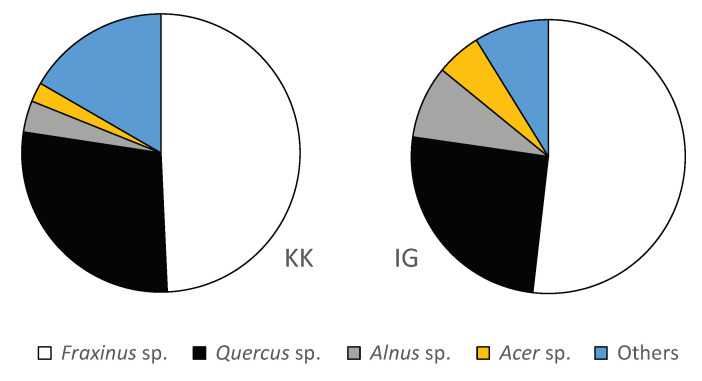
The relative abundance of the dominant taxa in the post assemblages per site cluster. (**a**) cluster Kamnik pod Krimom (KK), based on ten sites, *n* = 3578 posts, (**b**) cluster Ig, based on five sites, *n* = 2780 posts. Others = *Abies* sp., *Corylus* sp., *Carpinus* sp., *Fagus* sp., *Populus* sp., *Salix* sp., *Ulmus* sp., and a few samples that were not identified due to poor preservation.

Comparison of the taxon diversity for the overall time period indicates that the wood assemblage from both site clusters—as reflected by the posts from the pile dwellings—is mostly comparable. The same taxa occur both in cluster KK and IG in substantial numbers (Table 2). In both clusters, c. 50% of the posts are ash and 25% are oak (Figure 3 and Table 1). In the IG cluster, alder and maple reach values of 9% and 5% respectively. The remaining taxa in both clusters individually represent <5%. Alder appears to be more common in cluster IG (9%) than in cluster KK (4%), which is primarily caused by 9% alder at Parte-Iščica. Conversely, willow is better represented in cluster KK (5%) than in cluster IG (0.3%), caused by 9% willow at Založnica.

### 3.2. Diameter Analysis and the Splitting of Posts

**Figure 4:**
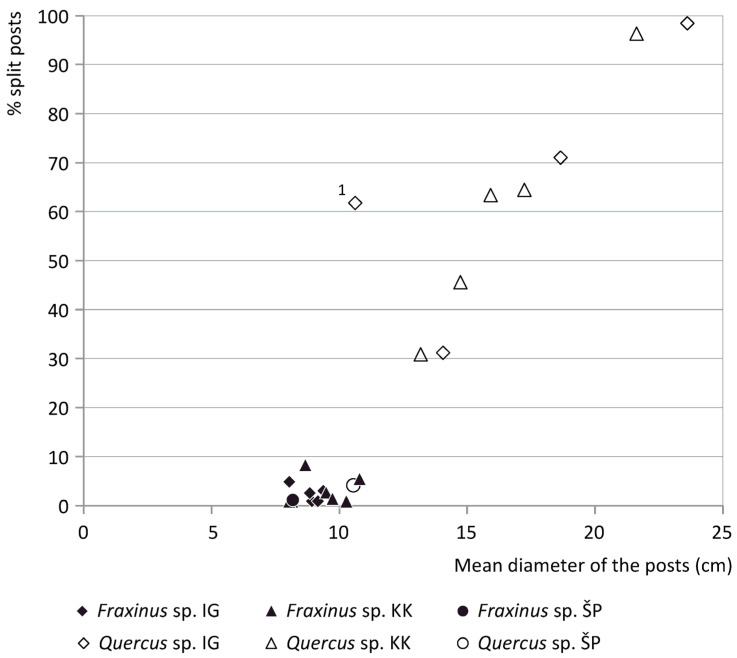
Scatterplot showing the percentage of split posts in relation to the mean diameter for ash and oak. Each data point represents a site. The dataset includes all sites with >50 posts with diameter data as well as oak data from Črnelnik (*n* = 27). 1 = Maharski prekop. IG = cluster Ig, KK = cluster Kamnik pod Krimom, ŠP = Špica.

Figure 4 shows the percentage of split oak and ash posts in relation to the mean diameter of the posts per site. The mean diameter of ash posts from sites varies between 8.1–11.2 cm (see Table S2 for the minimum and maximum values, with maximum values ranging between 12–35 cm). The ash posts are rarely split (max. 8% per site, Črnelnik (*n* = 10) excluded).

The diameter data of the oak post assemblage per site differs from those of ash. Firstly, the mean diameter of posts of oak varies between 10.3 and 23.6 cm per site, with maximum values between 11 and 64 cm, thus being larger than that of the ash posts. In addition, the percentage of split oak posts per site varies between 31–98%. Finally, there is a trend that the mean diameter and the percentage of split posts of oak are correlated: the larger the diameter, the higher the percentage of split posts. The data do not reveal clear differences between the regions or diachronic patterns.

There is one site—Maharski prekop—that is an outlier in Figure 4. Here, the percentage of split posts is larger than expected based on the mean diameter of the posts, and the mean diameter of the oak posts (11 cm) matches the common mean diameter of ash posts. It could be that oaks were split to obtain posts with dimensions similar to the ash trunks (more often than at other sites).

**Figure 5:**
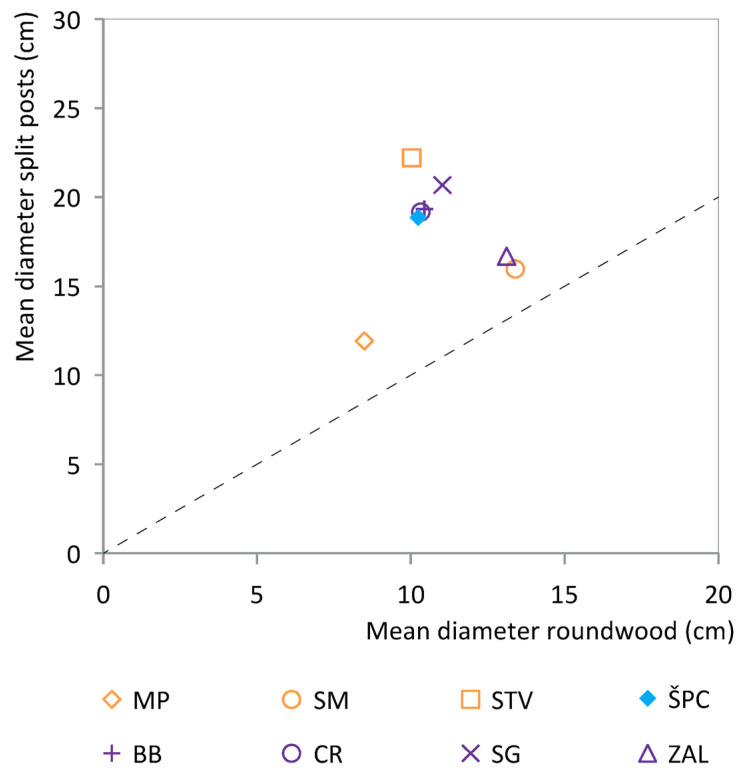
Scatterplot showing the mean diameter of split oak posts in relation to the mean diameter of unsplit oak posts. Each data point represents a site (see Table 1 for the explanation of the site name abbreviations). The dataset includes all sites with >50 posts with diameter data and with substantial numbers of both split and unsplit posts.

Figure 5 shows how the mean diameter of split and non-split oak posts relate to each other per site. The mean diameter of unsplit oak posts is always smaller than the mean value of split posts from the same site. The relation between the mean diameter of split and unsplit oaks differs between the sites. At Blatna Brezovica, Črešnja pri Bistri, Stare gmajne, Strojanova voda and Špica, the mean diameter of split trunks differs more from the mean diameter of roundwood than at Maharski prekop, Spodnje Mostišče and Založnica. These differences between sites, although subtle, may indicate different composition and characteristics of tree stands or differential exploitation, potentially because of different tool use. The data do not reveal clear differences between the regions or diachronic patterns.

### 3.3. Woodland Management

The presentation of the results on woodland management below focusses on a selection of primarily oak and ash posts from a few early and late sites from both geographic clusters with data representative of the results from all sites (for further details of the data from these and other sites see Supplementary Table S2 and Figure S1). In addition to the selected representative sites, a few exceptions to the general pattern will be highlighted.

**Figure 6:**
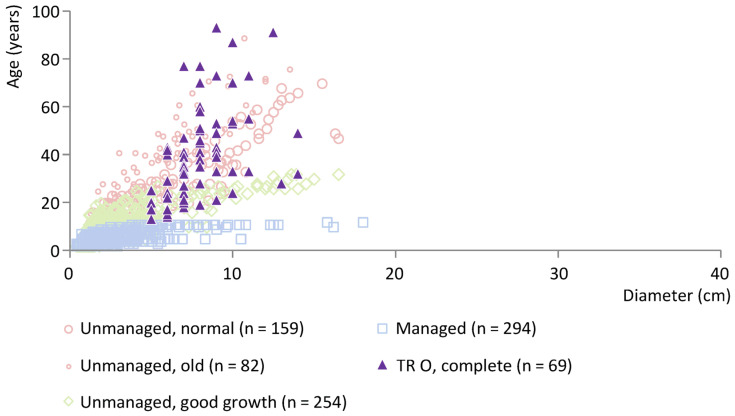
Ash posts from Trebež, cluster KK, plotted against a representative selection of modern ash reference data. O = roundwood.

Figure 6 shows the wood age/diameter data for posts of ash (*n* = 69) from Trebež, a relatively early site in cluster KK (end date 3649 BCE). The archaeological data are plotted against a selection of modern ash reference data (see Section 2.2). For all ash posts from this site, information about the pith and waney edge is available and so the posts are classified as complete. Information about whether it concerns roundwood or split wood is available for all ash posts except two (see Table S2 for further details). The data have a broad age range and primarily show an overlap with modern normally and fast-growing unmanaged ash trees, thereby indicating the use of unmanaged trees. Oak was also used at Trebež. Like the ash posts, the data of the oak posts, which are mostly split, point to the use of unmanaged woods (see Figure S1.2), but the number of posts retrieved (*n* = 14) is too small to draw robust conclusions.

**Figure 7:**
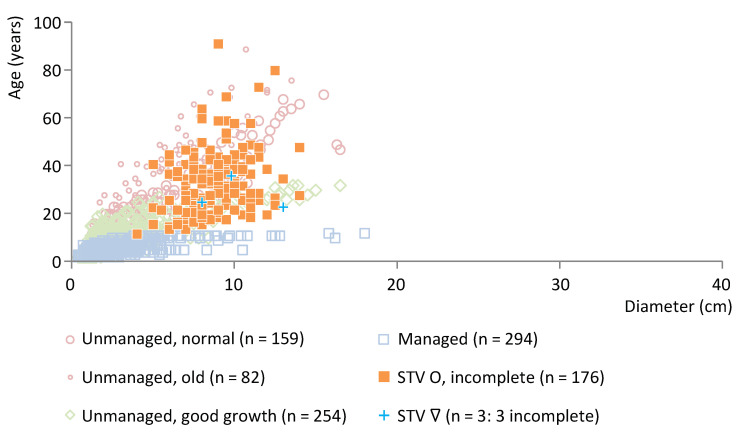
Ash posts from Strojanova voda, cluster IG, plotted against a representative selection of modern ash reference data. O = roundwood, ∇ = split wood.

A relatively early site in cluster IG is Strojanova voda (end date 3578 BCE). Figure 7 shows this site’s wood age/diameter data for ash posts (*n* = 179). For both ash and oak posts from this site no information about the presence of the pith was available and, as a result, the posts are classified as incomplete (see Table S2 and Section 2.1). Ninety-eight percent of the ash posts represent incomplete roundwood, while three posts are incomplete split posts (see Table S2). Similar to Trebež, the data have a broad age range and primarily show an overlap with modern normally and fast-growing unmanaged ash trees, thereby indicating the use of unmanaged trees.

If any years are missing since the posts are classified as incomplete, the dataset from Strojanova voda would reach older ages and larger diameters, thus moving more into the direction of unmanaged wood (and the same is true for other datasets of incomplete wood below). However, the classification as incomplete may also result from a lack of information rather than missing annual rings (see Section 2.1 and Table S2 for the details of all datasets).

Figure S1.2 shows the wood age/diameter data for oak posts from Strojanova voda (*n* = 124), plotted against modern oak reference data. The posts include incomplete roundwood (29%) and, other than ash, especially incomplete split wood (71%). The data have a broad age range and primarily show an overlap with modern average-aged oak trees growing under normal conditions and old unmanaged oak trees, thereby again indicating the use of unmanaged trees. Some additional results can be observed. Some of the oak posts, particularly those <40 years and both including roundwood and split wood, can be interpreted as fast-growing trees.

**Figure 8:**
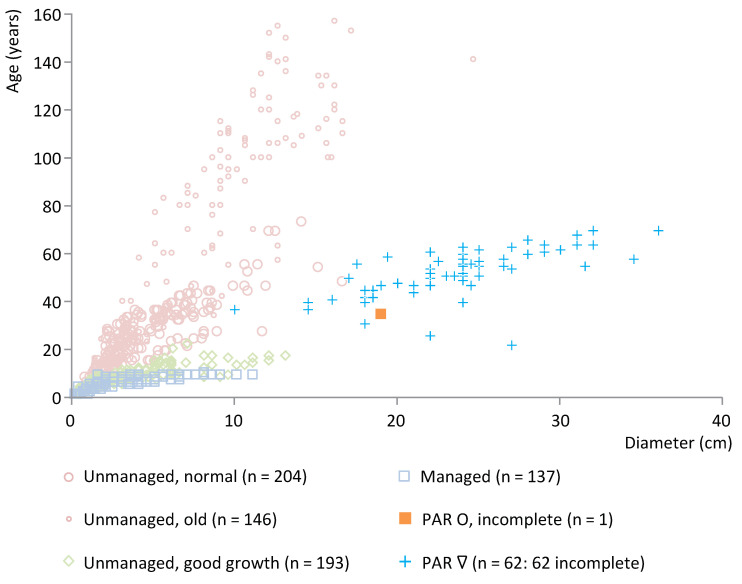
Posts of oak from Parte, cluster IG, plotted against a representative selection of modern oak reference data. O = roundwood, ∇ = split wood.

Parte is a relatively late site in IG (end date 2458 ± 18 BCE). Figure 8 shows the age/diameter data for the oak posts at this site (*n* = 63). With one exception, all these posts are split. Based on the absence of information about the presence of the pith, they have been categorized as incomplete split wood. These split oak posts plot outside the range of the modern data and can therefore not be compared directly but show a wide age range and the age increases with diameter, and thus most likely indicate the use of wood from unmanaged trees. The maximum age is relatively low. Figure S1.1 shows the wood age/diameter data for ash posts from Parte (*n* = 146). Based on the available information, all posts are classified as incomplete wood. The data show a wide age range and overlap particularly with roundwood of unmanaged trees, thereby indicating the use of unmanaged trees.

**Figure 9:**
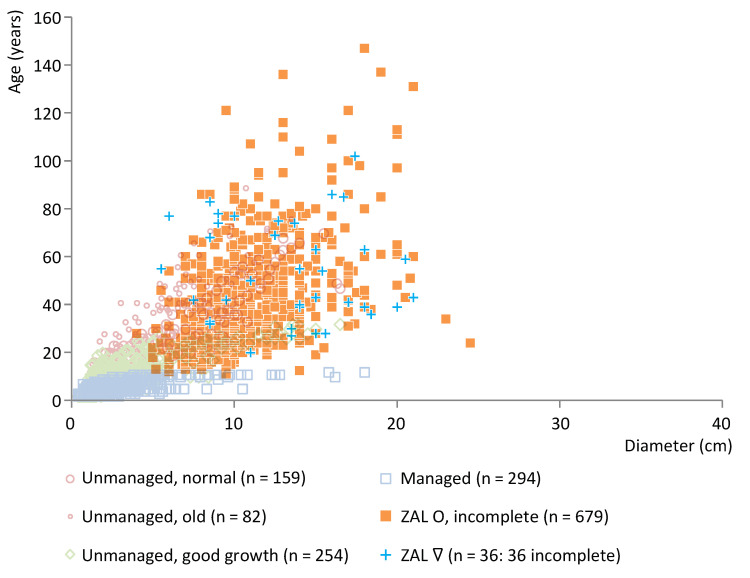
Posts of ash from Založnica, cluster KK, plotted against a representative selection of modern ash reference data. O = roundwood, ∇ = split wood.

A relatively late site in KK is Založnica (end date 2417 ± 18 BCE), located c. five km east of the other sites in this cluster, closer to IG compared to the other sites in KK. Figure 9 shows the age/diameter data for the ash posts (*n* = 715). Based on the available information, 95% of the posts are classified as incomplete roundwood while 5% concern split posts (only for these latter was it explicitly defined whether they were split or not). The data of the ash posts from Založnica show a wide age range and partially overlap with the roundwood of normally and fast-growing trees as well as old unmanaged trees, thereby indicating the use of wood from unmanaged vegetation. The data of the roundwood and split posts overlap.

**Figure 10:**
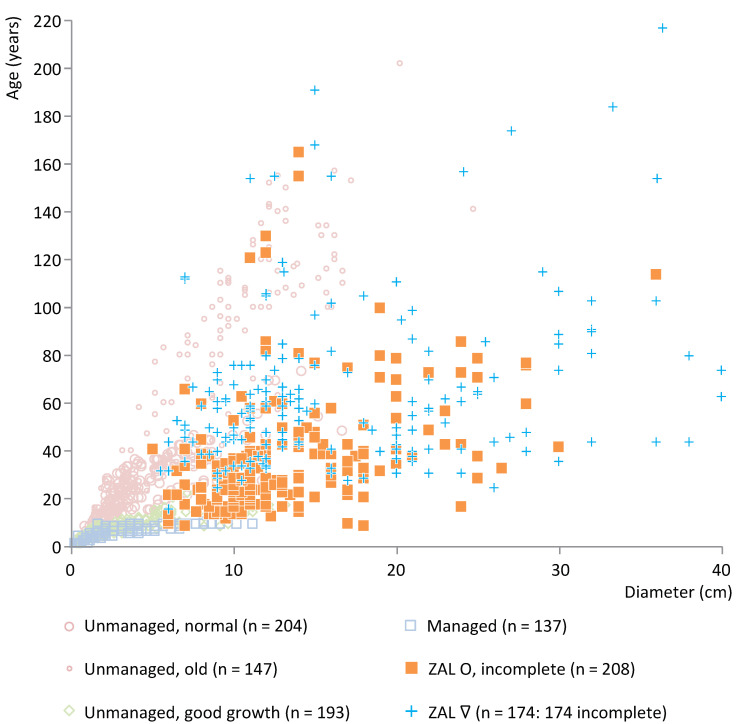
Oak posts from Založnica, cluster KK, plotted against a representative selection of modern oak reference data. O = roundwood, ߜ = split wood.

Figure 10 shows the age/diameter data for the oak posts from Založnica (*n* = 382). Based on the available information, 54% of the posts concern incomplete roundwood, while 46% concern incomplete split wood (see the note about incomplete wood in the description of Figure 7). Some of the Založnica oak posts plot outside the range of the modern reference data, but overall the data show a wide age range and seem to overlap primarily with roundwood of unmanaged trees, thereby indicating the use of wood from unmanaged trees. Few data overlap with the roundwood of managed trees.

In both the cases of ash and oak, some archaeological posts at Založnica plot in the overlap area of modern fast growing unmanaged trees and managed trees. The same is observed for ash posts at Maharski prekop, Dušanovo and Špica and oak posts at Špica (see Figure S1).

The age/diameter data of the willow posts from Špica (*n* = 55) and Založnica (*n* = 134) overlap with the roundwood of unmanaged trees (Figure S1.3), confirming the results of the ash and oak posts.

## 4. Discussion

### 4.1. Woodland Exploitation

The diversity of wood taxa used for posts in the two geographical clusters in Eneolithic Ljubljansko barje indicates that people had access to a wide range of taxa, including oak, ash, alder, maple, willow, poplar, beech, hornbeam, elm, hazel and silver fir. There are differences in the relative importance of the various taxa per site and over time, but the variable number of posts per site and the number of sites in relation to the long time period investigated hamper detailed comparisons.

The diversity of taxa attested in the post assemblages indicates rather opportunistic use of wood based on the availability of taxa. However, there are also arguments in favor of the selective use of construction wood. Firstly, the selection of construction wood is indicated by the fact that, in both clusters, c. 50% and 25% of the posts were of ash and oak, respectively. These taxa are both very suitable as construction wood because they have the most favorable mechanical properties (strength) of those taxa that were used as posts, while oak is also the only taxon with durable heartwood, under both waterlogged and dry conditions. Whether ash was preferred over oak or was more commonly available is not clear. The selection of ash is supported by the fact that it is not at all dominant in the pollen diagrams, e.g., [11,21,34,35,36,37,38]. Ash’s limited pollen production alone cannot explain this difference. Oak is frequently more common in the pollen diagrams than ash due to its higher pollen production, but selective use of oak is nevertheless supported by the fact that oak is much better represented in the overall post assemblage than, for example, beech, hazel and alder, which, according to the pollen diagrams, were also common in the vegetation. In addition, the use of oak for posts may have been avoided to conserve the wood for the production of artefacts, for its nuts and/or for cultural reasons.

In addition to the selective use of ash and oak, a comparison with the macrobotanical assemblage available from Črnelnik, Stare gmajne and Hočevarica [21,22] shows that various shrubs and small trees such as elderberry (*Sambucus* sp.), dogwood, Cornelian cherry, hawthorn (*Crataegus monogyna*), sloe (*Prunus spinosa*) and apple were not used as construction wood. The fruits of these taxa were probably more important to people as a food supply. Moreover, the trees of these taxa often grow in shapes that make the trunks unsuitable as construction wood. This avoidance of taxa also indicates the selective use of wood for the posts.

### 4.2. Diameter Selection and Splitting of Posts

The diameter analysis (Figure 4) shows that the ash posts have a mean diameter of 8.1–11.2 cm per site and mostly represent roundwood (max. 8% split posts per site), while the oak posts reach larger mean diameters per site of up to 23.6 cm and are much more often split (up to 98% per site). The differential quantity of split posts can be explained by the different mean diameter of the ash and oak posts. Oak trunks with a large diameter were apparently regularly split, possibly to obtain as many posts from a single trunk as possible and/or to obtain posts of more or less equal dimensions. Moreover, posts with a small transverse section are much easier to drive into the ground than larger ones, even more so when considering the substantial length of the pile dwelling posts (up to 8 m).

The mean diameter of unsplit oak posts from the sites is smaller than the mean diameter of split posts from the same sites (Figure 5). How the mean diameters of split and unsplit oak posts relate to each other varies per site, which may indicate the differential availability of tree stands or different exploitation strategies at each site. The differences do not show any clear patterns.

How to explain the large differences between the mean diameter per site of the ash and oak posts? A first hypothesis is that the different diameters simply reflect naturally available tree sizes for ash and oak. Although ash can easily naturally reach diameters up to 1–1.5 m [39,40], perhaps ash and oak grew at different locations (lakeshore versus hills) and were hence differentially affected by environmental conditions. An alternative hypothesis is that the ash wood was obtained by coppicing. However, the age/diameter analyses do not support this. Another alternative scenario is that the differential diameter of ash and oak indicates that people applied diameter selection, preferring ash posts of particular diameters. Such diameter selection could reflect a combination of the need for posts with a certain diameter combined with efforts to minimize the energy spent on felling, transporting and splitting the trees. If true, it remains a question why diameter selection was applied only to ash and not in a similar way to oak. Preference for thick oak trunks with relatively plentiful heartwood may have played a role. In conclusion, the different mean diameters of ash and oak posts are not yet completely understood.

### 4.3. Woodland Management

The comparison of the age/diameter data of both ash and oak posts with a large quantity of similar measurements of modern managed and unmanaged trees does not provide any substantial, indisputable evidence of woodland management for any of the investigated archaeological sites. Instead, most data indicate the exploitation of unmanaged vegetation. This result is the same for both site clusters KK and IG and was found at all sites of all periods, and is also supported by willow data from two sites. Generally, the results indicate that the way people exploited the woodlands in the Ljubljansko barje remained very stable over a period of at least c. 1300 years. Even if future investigations at a finer scale, such as per house structure or occupation phase, make a more convincing case for woodland management, the results here are sound because of the large number of posts and sites that were studied.

Interestingly, at a few—mostly late—sites, some age/diameter data of archaeological posts show overlap with the partially overlapping data sets of modern fast-growing and/or managed trees. At Založnica and Špica, some posts of both ash and oak show overlap with fast-growing unmanaged and/or normally growing managed trees, while the same is observed for ash posts at Maharski prekop and Dušanovo. Maharski prekop concerns a single site in the mid-4th millennium BCE and the relevant number of posts is very small (*n* = 12), hampering meaningful discussion. The three other sites, however, all date to the mid-3rd millennium BCE at the end of the occupation period on Ljubljansko barje, and the number of posts showing overlap or potential overlap with managed trees is larger (see Figure S1). On the one hand, the indications for fast-growing trees in the past implies that part of the data sets of these three late sites possibly represent managed trees. These sites were in use at the end of a longer occupation period, which may explain a change in vegetation exploitation. On the other hand, at least for ash, the work on modern trees clearly shows that unmanaged trees under good growing conditions may grow so fast that overlap with managed trees arises, resulting in similar age/diameter values (see Section 2.2). Overall, considering that the majority of the data from the three relevant sites show a wide age distribution pointing to the absence of management, the most likely interpretation of the data remains that there is no solid evidence for woodland management on a substantial scale. Use of not only unmanaged but also a small quantity of managed trees is not impossible (see also the further discussion below) but should be demonstrated by means of more unequivocal evidence.

The attested absence of evidence for woodland management is in some ways unexpected, since prehistoric people were very knowledgeable about tree growth and the resprouting of trees of many taxa after clearance. Moreover, oak and ash wood with wide annual rings—as found in managed trees—is also stronger than wood with narrow annual rings [41] (p. 89, 97). One possible reason for the use of unmanaged woodland is that there was no need to practice woodland management since there was sufficient wood available. This hypothesis is not supported by the pollen diagrams from Ljubljansko barje that show a decrease of arboreal taxa such as silver fir, beech and oak in the occupation horizons (see pollen references above). People may, however, have solved this by exploiting the woodlands in the nearby hills that are probably less well-represented in the pollen diagrams.

Another hypothesis is that woodland management was not practiced or could not be practiced because of factors such as herbivory by, e.g., beavers and possibly deer, as well as mobility and site function [7,8]. Particularly, site function and associated seasonality aspects have recently been raised as relevant for understanding woodland management practices at Circum-Alpine lakeshore settlements, with seasonal instead of year-round occupation explaining the absence of woodland management [42]. At Ljubljansko barje, however, with clear evidence that the sites are habitation sites rather than special activity sites, temporary occupation is not considered a plausible explanation for the absence of management, since there are indications of occupation during both summer and autumn/winter (see Section 1).

Another possible explanation for the lack of evidence of woodland management is that it was practiced but remains undetected. One underlying mechanism could be that while woodland management was practiced, the resulting wood was not used for posts. Although the macroremains assemblages do not provide concrete indications of use other than the usual exploitation of all kinds of plant resources, both oak and ash equally may have been managed to obtain optimal harvests of acorns and leaf fodder, respectively, instead of timber. This may have resulted in wood growth that does not match growth patterns as expected in the case of formal management for wood harvest (i.e., clearance of branches from multiple trees, e.g., every seventh year). It may also have been other taxa, e.g., willow, hazel and alder, that were managed for purposes such as wattle work, leaf fodder and/or fuel. However, two analyzed age/diameter data sets of willow from Špica and Založnica, although representing a small sample, do not support this hypothesis.

Alternatively, it is possible that trees were managed but that this is currently not recognized because of long cutting intervals. Investigation of modern trees has shown that the fast growth of managed ash decreases after c. 15 years [32,43] and that the age/diameter data of managed ash trees, when cut 30–50 years ago, overlap completely with those of unmanaged trees. This may theoretically also be the case for the posts from Ljubljansko barje, since the mean age of ash posts ranges between 26 and 50 years per site, with maximum values of up to 156 years. For oak, it is difficult to evaluate this hypothesis since the reference data set is still small.

Another possible explanation for the invisibility of woodland management is that if woodland management was practiced, it was applied in a much less comprehensive way than nowadays or as described in historical sources (adventitious management [8] (Figure 8) and the related discussion there, after [44,45]). This is difficult to recognize by age/diameter analysis since the method is based on the reference data of formal coppice, in which multiple trees are subjected to the same management regime. In addition, possible trends may be even more difficult to detect since the archaeological posts that were subjected to age/diameter analysis represent only a selection of the material from the sites (see Section 2.1), of which it is not precisely clear how they contextually relate to each other (e.g., whether they form structures and/or belong to different structures). Repeated incidental wood collection in the Eneolithic could possibly explain the indications of the exploitation of fast-growing trees at Založnica and Špica, two sites that date to the end of a longer occupation phase. While this may have been the case, formal management is not proven and most of the data from these sites indicates the absence of management.

How does the lack of evidence of woodland management at Ljubljansko barje relate to other comparable sites? One the one hand, the lack of evidence of woodland management matches the absence of evidence at Mesolithic and Neolithic sites in other countries in Europe, including England, Spain, the Netherlands and Denmark [7,8,9,44]. On the other hand, the result is in apparent contrast to dendrotypological studies about woodland exploitation in contemporaneous German and Swiss lake shore settlements, where research indicates the practice of woodland management (see e.g., [3,42] and Section 1.1). How to explain this difference? This question is currently a topic of debate on its own [42,46]. Rather than analyzing wood with different diameters, it seems that dendrotypology detects adventitious management while age/diameter analysis does not, as explained above. If adventitious management is expected at any of the studied Slovenian sites, it is at the sites at the end of the studied period and perhaps towards the end of the periods 3700–3330 BCE and 3160–3070 BCE.

In conclusion, for the first time, this paper applies age/diameter analysis to posts from multiple related archaeological sites using two geographical clusters c. 10 km apart in Ljubljansko barje in Eneolithic Slovenia as a case study. The data at all sites give very uniform results, both concerning taxon variety and woodland management, showing that woodland exploitation in the two studied areas was very similar. The ash trunks have a remarkably small and uniform mean diameter per site compared to the oak trunks, something which may relate to availability and/or diameter selection. The data do not provide unequivocal evidence of woodland management and indicate instead that people most likely primarily, if not exclusively, exploited unmanaged woodlands, thus mainly indicating continuity. Only at the end of the studied period is there evidence of the exploitation of faster-growing trees. People may have started to use wood from the same trees repeatedly, although in a rather opportunistic way. Suggestions for future research include the analysis of other, similarly large datasets of pile dwellings, also including contextualization of the wood (i.e., linking posts to possible structures), where the available information permits. Additionally, it remains important to continue research on woodland management, collect data from other sites and regions, and include data from younger periods.

## Figures and Tables

**Table 1:** The studied sites with information about the number of posts, the quantity of posts of ash (*Fraxinus* sp.) and oak (*Quercus* sp.), and the excavation year. Cl. = geographical cluster, KK = Kamnik pod Krimom; IG = Ig; *n* = number. 9 (10) = 9 posts with age and diameter data; 10 posts in total of which there is one for which no diameter and/or age data are available. Črni graben and Dušanovo represent two excavations of what has been later interpreted as a single site, Dušanovo.

Site	Code	Cl.	End Date (Years cal BCE)	Posts Total (*n*)	Posts Ash (*n*)	Posts Oak (*n*)	Posts Ash (%)	Posts Oak (%)	Excavation Year(s)
Črnelnik	CEN	KK	3694	50	9 (10)	27 (28)	20	56	2014
Trebež	TR	KK	3649	115	69 (70)	17 (21)	61	18	2017
Strojanova voda	STV	IG	3578	354	179	124 (131)	51	37	2012
Hočevarica	HOC	KK	3570	350	196 (213)	48 (58)	61	17	1995, 1998
Maharski prekop	MP	IG	3487	234	69	81 (82)	29	35	2005
Črešnja pri Bistri	CR	KK	3407	123	25	60 (61)	20	50	2003
Spodnje Mostišče	SM	IG	3351	686	140 (152)	320 (403)	22	59	1996, 1997
Stare gmajne	SG	KK	3109 ± 14	932	397 (409)	323 (334)	44	36	2002, 2004, 2006, 2007
Veliki Otavnik	VO	KK	3108 ± 14	30	6	17	20	57	2006
Blatna Brezovica	BB	KK	3071 ± 14	178	55	94 (95)	31	53	2003
Parte-Iščica	PI	IG	c. 2610	1265	802 (896)	17 (25)	71	2	1997, 1998
Črni graben	CG	KK	2491 ± 18	50	50	-	100	0	2010
Parte	PAR	IG	2458 ± 18	241	146 (154)	63 (76)	64	32	1996
Založnica	ZAL	KK	2417 ± 18	1479	715 (723)	382 (388)	49	26	1995, 1999, 2001, 2009, 2015
Dušanovo	DU	KK	c. 2450	271	202	5	75	2	2010, 2013, 2017
Špica	SPC		c. 2450	2452	1287 (1460)	381 (509)	60	21	2009, 2010, 2011

**Table 2:** The post wood identifications of the four dominant taxa for each of the studied sites in %. The sites are chronologically ordered (see Table 1). Cl.= cluster, KK = Kamnik pod Krimom, IG = Ig; Others = *Abies* sp., *Corylus* sp., *Carpinus* sp., *Fagus* sp., *Populus* sp., *Salix* sp., *Ulmus* sp. and a few samples that were not identified due to poor preservation.

Site	Cl.	End Date (Years cal BCE)	n_total_ Posts	*Acer* sp. (%)	*Alnus* sp. (%)	*Fraxinus* sp. (%)	*Quercus* sp. (%)	Others (%)
Črnelnik	KK	3694	50	0	0	20.0	56.0	24.0
Trebež	KK	3649	115	0	18.3	60.9	18.3	2.6
Strojanova voda	IG	3578	354	2.8	7.3	50.6	37.0	2.3
Hočevarica	KK	3570	350	2.3	9.4	60.9	16.6	10.9
Maharski prekop	IG	3487	234	10.3	11.5	29.5	35.0	13.7
Črešnja pri Bistri	KK	3407	123	1.6	22.0	20.3	49.6	6.5
Spodnje Mostišče	IG	3351	686	11.4	6.1	22.2	58.7	1.6
Stare gmajne	KK	3109 ± 14	932	3.2	3.3	43.9	35.8	13.7
Veliki Otavnik	KK	3108 ± 14	30	0	6.7	20.0	56.7	16.7
Blatna Brezovica	KK	3071 ± 14	178	8.4	1.7	30.9	53.4	5.6
Parte-Iščica	IG	c. 2610	1265	2.8	9.4	70.8	2.0	15.0
Črni graben	KK	2491 ± 18	50	0	0	100.0	0	0
Parte	IG	2458 ± 18	241	0.4	4.1	63.9	31.5	0
Založnica	KK	2417 ± 18	1479	1.8	0.9	48.9	26.2	22.2
Dušanovo	KK	c. 2450	271	0	0	74.5	1.8	23.6
Špica		c. 2450	2451	3.1	2.0	59.6	20.8	14.5

## Data Availability

The complete data sets of modern ash and willow are published in Out, W.A., Hänninen, K., Vermeeren, C. Using branch age and diameter to identify woodland management: new developments. *Environmental Archaeology*, **2018**, *23(3)*, 254–266. The data presented in the supplementary files of this study are openly available in the repository of the University of Ljubljana at [https://repozitorij.uni-lj.si/IzpisGradiva.php?id=142470].

## Supplementary Materials

The following supporting information can be downloaded at: https://www.mdpi.com/article/10.3390/plants12020291/s1. Table S1: The wood identifications for each of the studied sites in numbers. Table S2: Age and diameter data from modern reference trees of ash and oak and age and diameter data from the archaeological sites, sorted by taxon and site. Figure S1 Age and diameter scatterplots of posts of ash, oak and willow from the studied Eneolithic archeological sites in Slovenia (see Table 1 for an overview of the sites) as well as from modern trees. O = roundwood, ∇ = split wood.
